# Revisiting the isolation and characterisation of *Entamoeba histolytica* lipopeptidophosphoglycan

**DOI:** 10.1007/s00436-024-08149-6

**Published:** 2024-02-21

**Authors:** Anna Nagode, Jorick Vanbeselaere, Michael Duchêne

**Affiliations:** 1 Institute of Specific Prophylaxis and Tropical Medicine, Center for Pathophysiology, Infectiology and Immunology, Medical University of Vienna, Vienna, Austria; 2 Department of Chemistry, Universität für Bodenkultur Wien, Vienna, Austria

**Keywords:** *Entamoeba histolytica*, Lipopeptidophosphoglycan, Butanol, Extraction, Digestion, Scandium triflate

## Abstract

The parasite *Entamoeba histolytica* is the cause of amoebic dysentery and liver abscess in humans. On the protozoan cell surface, a variety of glycosylated molecules are involved in the interaction with the environment, such as attachment to the colonic mucus. One of these molecules is the lipopeptidophosphoglycan (LPPG), a complex surface component with antigenic properties. Its structure is only partly known, it is a glycosylphosphatidylinositol (GPI)-linked glycoprotein with a large amount of O-glycosylation. To date, the sequence of a core protein has not been identified. In this study, we further investigated this complex surface molecule aided by the availability of the monoclonal antibody EH5, which had been raised in our laboratory. We studied the extraction of LPPG in various solvent mixtures and discovered that 2-butanol saturated water was simple and superior to other solvents used in the past. The isolated LPPG was subjected to treatment with several proteases and the Ser/Thr specific cleavage agent scandium (III) trifluoromethanesulfonate (scandium triflate). The products were probed with antibody EH5 and the blots showed that the LPPG preparation was largely resistant to standard proteases, but could be cleaved by the scandium compound. These observations could point to the existence of a Ser- or Thr-rich core protein structure.

## Introduction

*Entamoeba histolytica*, a protozoan parasite, is the causative agent of amoebic colitis and amoebic liver abscess in humans. *E. histolytica* trophozoites inhabit the lumen of the colon feeding on microbiota and glycans of the outer mucus layer (Leon-Coria et al. 2020). The parasite interacts with the enteric environment via various surface molecules, such as glycoproteins or proteophosphoglycans. The major and best studied surface protein is the Gal/GalNAc lectin, a complex of a heavy, light and intermediate subunit binding to galactose and *N*-acetylgalactosamine (GalNAc) of the human mucus and enterocytes (Frederick and Petri 2005). It constitutes the main player in adhesion and signal transduction (Vines et al. 1998) and LecA, a 578 residue fragment of the heavy chain, was investigated as a candidate for a vaccine against *E. histolytica* (Barroso et al. 2014).

A further important surface molecule and the focus of this study is the lipopeptidophosphoglycan (LPPG). It was originally isolated and localised by Isibasi et al. (Isibasi et al. 1982a, b). Subsequently, monoclonal antibodies against *E. histolytica* glycoconjugates were developed. In one study, the material was reported as resistant to pronase treatment (Prasad et al. 1992), in another study (Stanley et al. 1992) the material was sensitive to pronase treatment, showing the uncertainty about the absence or presence of a peptide component. Further, it was discovered that LPPG was highly expressed in axenic amoebic strains but not, or very little, in strains with associated bacteria (Bhattacharya et al. 1992a), or in the less virulent *E. dispar* (Marinets et al. 1997; Bhattacharya et al. 2000). LPPG is not present in other *Entamoeba* species such as *E. moshkovskii* or *E. invadens* (Srivastava et al. 1995). In our lab, monoclonal antibodies were raised against a membrane fraction of *E. histolytica*. The antibody called EH5 was binding to LPPG, it was able to protect immunocompromised mice against amoebic liver abscess (Marinets et al. 1997), and human intestinal xenografts against inflammation and tissue damage (Zhang et al. 2002). Later, it was discovered with the help of mimotope libraries that EH5 bound to a motif with the consensus sequence Gly-Thr-His-Pro-X-Leu (Melzer et al. 2002). At the same time, Moody-Haupt et al. gave structural insights into the glycan parts of LPPG (in this study termed PPG), showing that LPPG is a glycosylphosphatidylinositol (GPI)-anchored protein with extensive glycosylation comprising dextran-like side chains that are O-linked to serine residues of the core protein via phosphodiester bonds and a core galactose residue (Moody-Haupt et al. 2000). Another unusual feature of the molecule is the presence of two forms of phosphatidylinositol *Eh*PIa and *Eh*PIb where *Eh*PIb carries an additional fatty acid modification on the inositol ring (Lotter et al. 2009). Surprisingly, until now, a polypeptide backbone of LPPG has not been identified.

Several immunological activities of LPPG were discovered, so LPPG can induce the formation of human neutrophil extracellular traps (Ávila et al. 2016). Moreover, a synthetic analogue of the phosphatidylinositol anchor of LPPG, *Eh*PIb, has immunostimulatory effects, which can be exploited to fight against *Leishmania* infections in vitro and in vivo (Choy et al. 2017; Fehling et al. 2020).

Isolation of LPPG from *E. histolytica* trophozoites was originally performed by hot phenol-water extraction (Isibasi et al. 1982a), but was also achieved by an optimised, multi-step chloroform–methanol-water extraction followed by treatment with solvent E, a solvent composed of water, ethanol, diethyl ether, pyridine and ammonium hydroxide (Bhattacharya et al. 1992b). More recent studies combined an ultracentrifugation step with chloroform–methanol-water delipidation and a subsequent phenol-water extraction for LPPG isolation (Lotter et al. 2009; Ávila et al. 2016). Those isolation methods are lengthy and require the usage of several toxic chemicals.

In this study, we found that 2-butanol-saturated water was a suitable agent to extract LPPG from whole *E. histolytica* trophozoites. The extracted material was subjected to several proteases and a chemical agent with different preferential cleavage sites, and the products were examined in western blots using the antibody EH5. Only the Lewis acid scandium triflate completely destroyed the epitopes of LPPG. No peptide fragments suitable for mass spectrometry were generated.

## Materials and methods

### Parasite culture

*Entamoeba histolytica* HM-1:IMSS trophozoites were cultured anaerobically at 37 °C in TYI-S-33 medium (Diamond et al. 1978) supplemented with 10% (v/v) complement-inactivated, bovine serum, 1% (v/v) penicillin/streptomycin solution (10,000 units penicillin and 10 mg/ml streptomycin; Sigma-Aldrich, USA) and 3% (v/v) of vitamin mixture (Diamond Vitamin Tween 80 Solution, SAFC Biosciences, KA, USA). The trophozoites were sub-cultured 1:7 in 12.5 cm^2^ tissue culture flasks (Corning, USA) twice a week under sterile conditions.

### LPPG extraction with the solvent E

LPPG was extracted as described before (Prasad et al. 1992). Briefly, 1 × 10^6^ *E. histolytica* trophozoites were harvested 24 h after sub-culturing and washed twice with cold 1 × PBS. Subsequently, the cell suspension was centrifuged, and the resulting cell pellet was extracted with 1 ml chloroform/methanol (3:2) mixed with 200 µl of 4 mM MgCl_2_, then with 1 ml of chloroform/methanol/water (10:10:3) together with 1 ml of chloroform/methanol (1:1) and finally, with 1 ml chloroform/methanol/water (10:10:3). The resulting pellet was extracted with 1 ml of solvent E, a mixture of water, ethanol, diethyl ether, pyridine and ammonium hydroxide (15:15:5:1:0.017) (Turco et al. 1984). The resulting LPPG extract was dried in a vacuum concentrator. Extractions were performed at least twice in independent experiments.

### LPPG extraction by hot phenol-water solution

*E. histolytica* trophozoites (1 × 10^7^) were harvested 48 h after sub-culturing and washed twice with cold 1 × PBS. A 1:1 phenol-water solution (w/v) was prepared at 70 °C, in which the *E. histolytica* cells were incubated for 30 min. Subsequently, the preparation was cooled down to room temperature and centrifuged at 5500 × *g* for 30 min at 4 °C. Finally, the upper aqueous phase containing LPPG and glycans was collected and dialysed against 2-butanol-water (1:1) overnight at 4 °C before the extract was dried in a vacuum concentrator. Extractions were performed at least twice in independent experiments.

### LPPG extraction by alcohol-water solutions

**Fig. 1:**
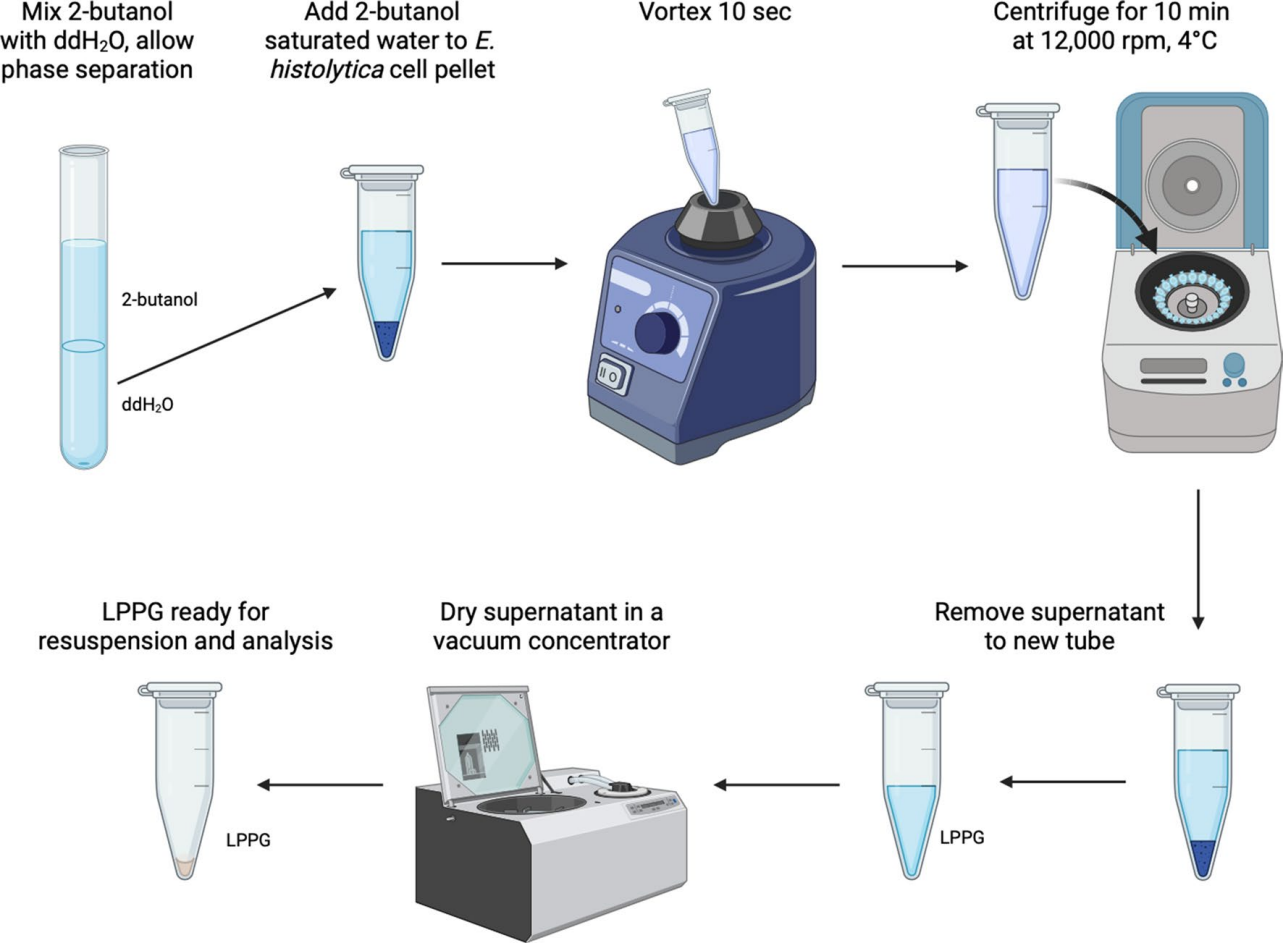
*E. histolytica* LPPG was extracted with water saturated either with 3-methyl-1-butanol, 2-methyl-1-propanol, 1-butanol, 2-butanol, or 1:1 (v/v) mixtures of the water-soluble alcohols 1-propanol or 2-propanol. Here the extraction with 2-butanol saturated water is shown, which turned out to be the best procedure. Water is saturated with 2-butanol and added to the pelleted *E. histolytica* trophozoites, vigorously mixed and centrifuged to separate LPPG from insoluble cell components. The supernatant is subsequently dried in a vacuum concentrator for western blot analysis

*E. histolytica* trophozoites were harvested 48 h after sub-culturing and washed twice with cold 1 × PBS. For each condition, 5 × 10^5^ trophozoites were pelleted and subsequently resuspended in 750 µl of the respective alcohol-water solution (Fig. 1). The alcohols and water were mixed 1:1 (v/v) at room temperature. In case of phase separation (C4 upwards), the lower aqueous phase was used. The cell suspension was vortexed for 10 s and centrifuged for 10 min at 12,000 rpm, 4 °C. The resulting supernatant was dried in a vacuum concentrator. Cell pellets and dried supernatant samples were resuspended in 1 × PBS and mixed with 4 × Laemmli sample buffer for SDS-PAGE and western blot analysis. Extractions were performed at least twice in independent experiments.

### SDS-PAGE and western blot

Each sample was separated on a 12.5% SDS–polyacrylamide gel. Protein transfer from the gel to a PVDF membrane (Bio-Rad) was achieved by using a wet blotting system. Following the transfer, the membrane was blocked in 3% (w/v) BSA and subsequently incubated in the primary antibody solution (1:20,000 dilution in 3% BSA) containing the mouse monoclonal EH5 antibody. The blots were washed three times in tris-buffered saline with Tween 20. The secondary alkaline phosphate-coupled goat anti-mouse antibody (Sigma, A3562) was then applied in a 1:10,000 dilution and finally, after washing as above, the blot was stained with 5-bromo-4-chloro-3-indolyl phosphate (BCIP) and nitro blue tetrazolium (NBT) in a buffer containing 100 mM Tris–HCl, pH 9.5, 100 mM NaCl and 5 mM MgCl_2_ for 5 min.

### Susceptibility of LPPG to proteases and scandium triflate

Investigation on the proteolytic and scandium triflate sensitivity of LPPG was carried out using the 2-butanol saturated water and the hot phenol-water extracts prepared as described above. The dried extracts were resuspended in 100 µl of the respective digestion buffer and incubated with 1 µg of each protease or 0.5–10 mM of scandium triflate. The product number, manufacturer of the various cleavage agents, as well as the digestion conditions and buffers are summarised in Table 1 below.

**Table 1:** Product number, manufacturers, digestion conditions and buffers for digestion of extracted LPPG. Digestions were performed at least twice in independent experiments

Compound	Product Nr	Digestion condition	Digestion buffer
Type I Trypsin from bovine pancreas	T8003, Sigma	2 h, 37 °C	50 mM Tris–HCl pH 8.0, 0.5 mM CaCl_2_
alpha-Chymotrypsin from bovine pancreas	C4129, Sigma	2 h, 37 °C	50 mM Tris–HCl pH 8.0, 0.5 mM CaCl_2_
Proteinase K from *Tritirachium album*	P2308, Sigma	2 h, 37 °C	50 mM Tris–HCl pH 8.0, 0.5 mM CaCl_2_
Thermolysin metalloproteinase	V4001, Promega	2 h, 75 °C	50 mM Tris–HCl pH 8.0, 0.5 mM CaCl_2_
Type XIII protease from *Aspergillus saitoi*	P2143, Sigma	2 h, 37 °C	50 mM glycine–HCl pH 2.8
Pronase from *Streptomyces griseus*	53702, Merck	16 h, 37 °C	50 mM K_2_HPO_4_, pH 7.5
Scandium triflate	483354, Sigma	2 h, 60 °C	50 mM Tris–HCl pH 8.0, 0.5 mM CaCl_2_

## Results

### LPPG can be extracted by a variety of alcohol-water solutions

**Fig. 2:**
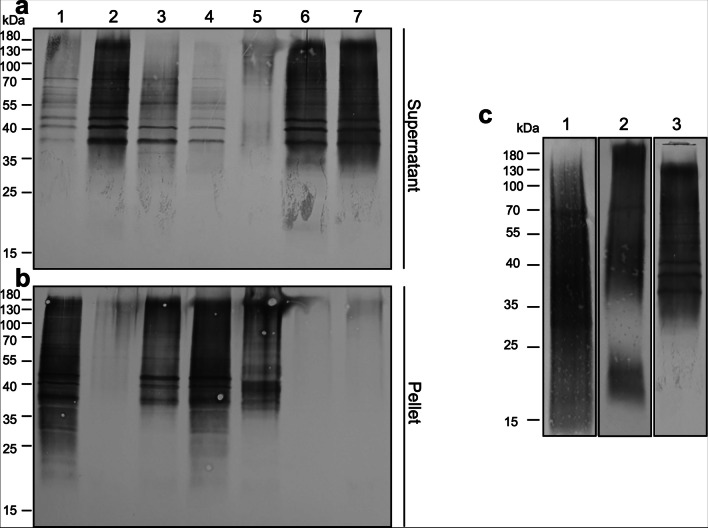
Extraction of LPPG from *E. histolytica* trophozoites. Comparison of alcohol-water (1:1) solutions for extraction of LPPG from 5 × 10^5^ *E. histolytica* trophozoites (**a**, **b**). The solutions were: water (1), water saturated with 2-methyl-1-propanol (2), water mixed 1:1 (v/v) with 1-propanol (3) or 2-propanol (4), water saturated with 3-methyl-1-butanol (5), 1-butanol (6), or 2-butanol (7). The solutions (1), (3), (4) and (5) were not sufficiently able to extract LPPG from the *E. histolytica* cells, resulting in EH5 signal in the pellet samples (**b**). The solutions of butanols (2), (6) and (7) completely solubilised LPPG from the amoebae, as virtually all the EH5 signal could be found in the supernatant (**a**). Comparison of three LPPG extraction methods (**c**): Delipidation with subsequent solvent E extraction (1), phenol-water extraction (2) and 2-butanol saturated water extraction (3). Western blot analysis was performed with the anti-LPPG EH5 monoclonal antibody

LPPG is less hydrophobic than many other membrane components but less hydrophilic than typical intracellular proteins. To develop a quick and easy extraction method for *E. histolytica* LPPG, various simple alcohol-water mixtures with graded hydrophobicity were put into consideration. Five × 10^5^ trophozoites were dissolved in water (1), with the aqueous phase of water mixed 1:1 of 2-methyl-1-propanol (isobutanol) (2), water mixed 1:1 with 1-propanol (3), or 2-propanol (4), or the aqueous phases of water mixed with 3-methyl-1-butanol (isoamylalcohol) (5), 1-butanol (6), or 2-butanol (7). Western blot analysis of the resulting supernatants and pellets samples with antibody EH5 (Fig. 2a, b) shows that LPPG was efficiently extracted from the amoebic membranes by 2-methyl-1-propanol, 1-butanol, and 2-butanol saturated water solutions. On the other hand, the solutions containing 1-propanol, 2-propanol, and 3-methyl-1-butanol solubilised LPPG from the trophozoite membranes very poorly and LPPG remained in the cell pellet (Fig. 2b). Water saturated with any of the three butanols was superior to any other solvents with marginal differences between the butanols. We then decided to use 2-butanol saturated water for the further investigations.

Figure 2c shows a comparison of two long-established LPPG extraction methods and the 2-butanol water LPPG extraction (lane 3). The 2-butanol saturated water results in a highly diffuse band from 30 to 180 kDa, whereas delipidation with subsequent extraction by solvent E generates an even more extended band from 15 to 130 kDa (lane 1). Lane 2 shows LPPG extracted by hot phenol-water, which exhibits a wide band similar to the 2-butanol saturated water extract from 35 to 190 kDa and a distinct additional band from 18 to 23 kDa.

Although the LPPG always migrated as a broad smear, the signals differed among the extraction methods. Of course, the LPPG is not a single molecule but contains molecules with varying numbers and lengths of the dextran-like side chains. The extraction methods may be more or less favourable for some of these. In addition, limited digestion may occur during the extraction procedures. This points again to the need for the identification of the core structure.

### LPPG exhibits resistance to various natural proteases but is sensitive to scandium triflate

**Fig. 3:**
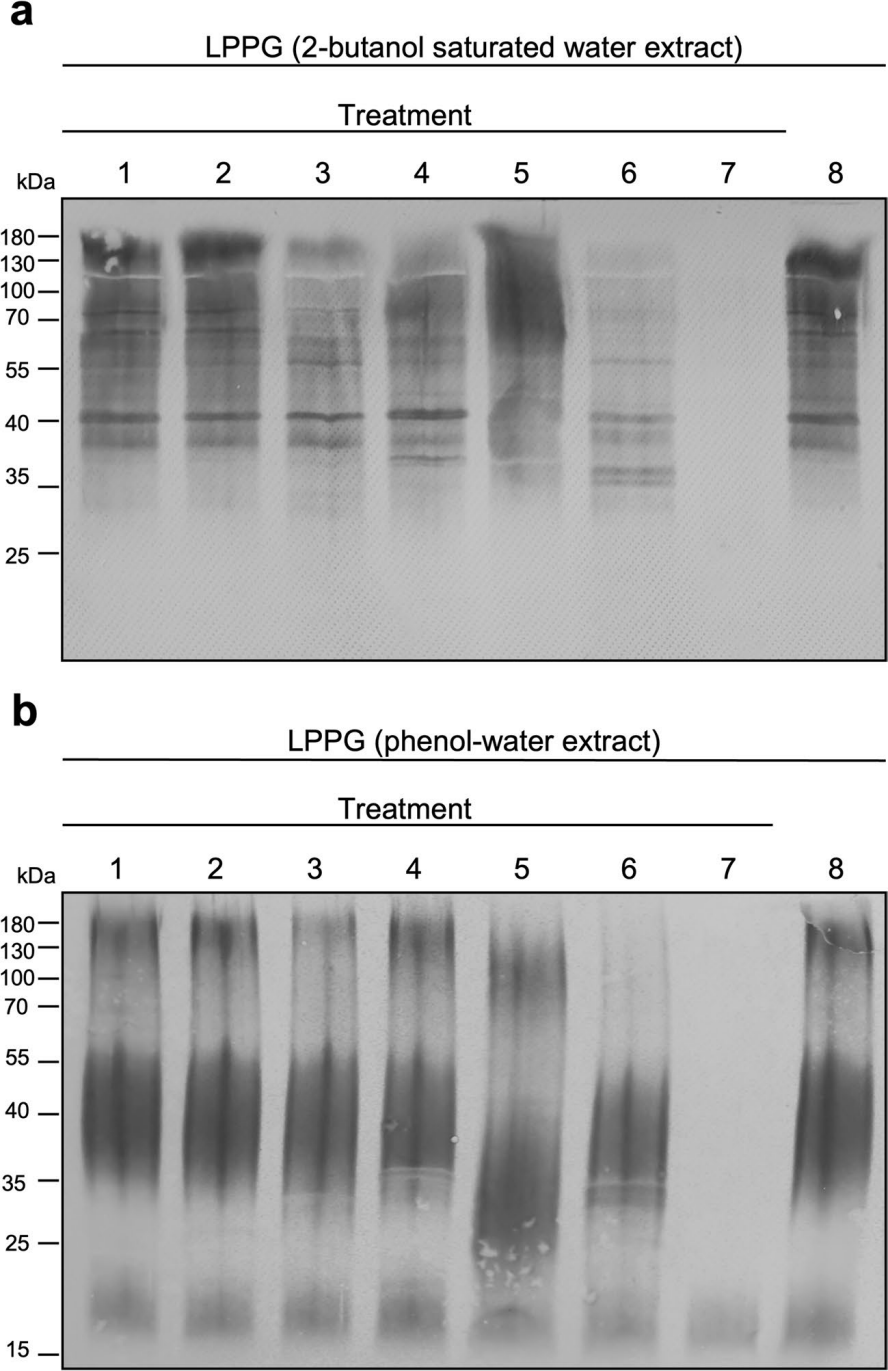
Exposure of LPPG to various cleavage agents. LPPG is considerably resistant to a variety of natural proteases trypsin (1), chymotrypsin (2), proteinase K (3), thermolysin (4), *Aspergillus* protease (5), and pronase (6), while it is degraded by the chemical cleavage agent scandium triflate (7). The untreated LPPG extract is shown in (8). The upper blot (**a**) shows the LPPG extracted by 2-butanol saturated water and the lower blot (**b**) the LPPG extracted by hot phenol-water. Western blot analysis was performed with the anti-LPPG EH5 monoclonal antibody

LPPG from *E. histolytica* trophozoites was extracted with 2-butanol saturated water or phenol-water. The extracts were subsequently incubated with trypsin (1), chymotrypsin (2), proteinase K (3), thermolysin (4), *Aspergillus* protease (5), pronase (6) or scandium triflate (7). The western blot analysis of extracts with 2-butanol-saturated water clearly shows that neither natural protease was able to fully degrade LPPG (Fig. 3a). Nevertheless, proteinase K and thermolysin slightly digested the bands around 100–180 kDa, and pronase strongly decreased the intensity of the signal over its whole length and additionally, led to the appearance of two bands slightly above 35 kDa.

LPPG extracted by phenol-water showed a band of about 130–180 kDa and another of 30–55 kDa, which reacted similar with the treatments (Fig. 3b). Trypsin, chymotrypsin and thermolysin did not change the appearance of LPPG on the western blot, while proteinase K slightly reduced the intensity of the 130–180 kDa band. *Aspergillus* protease led to a downward shift of both bands, while pronase completely cut the 130–180 kDa band. In contrast, both LPPG extracts could be efficiently cleaved by scandium triflate.

### Degradation of LPPG with scandium triflate

**Fig. 4:**
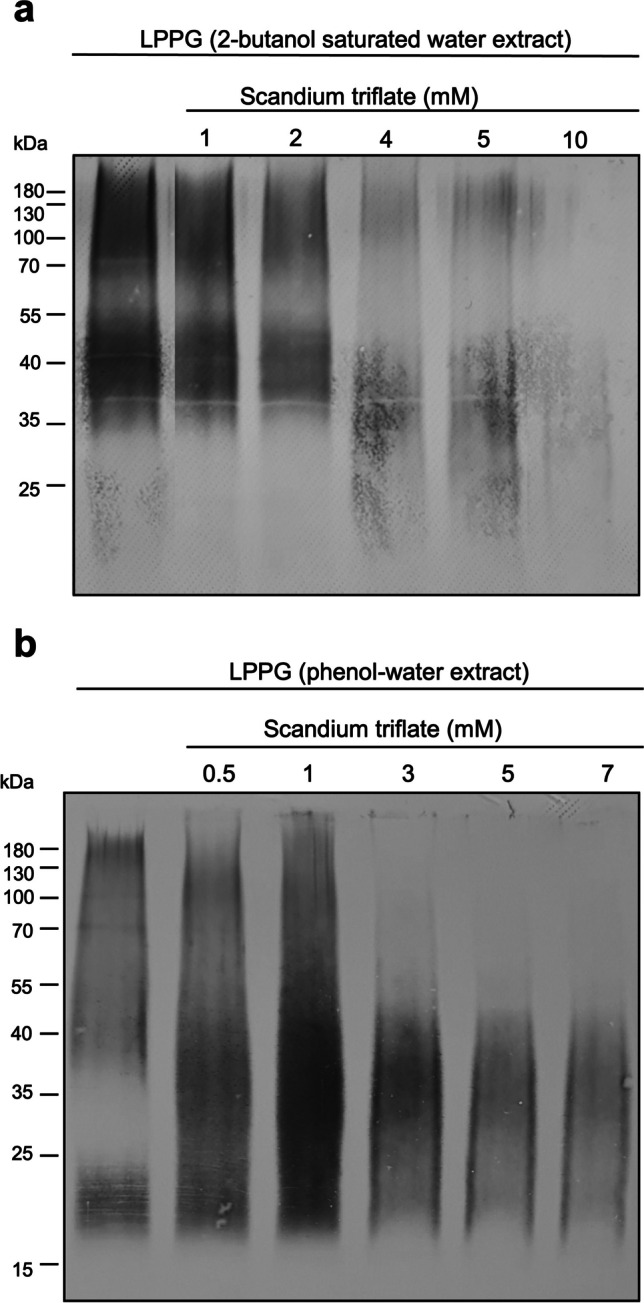
Cleavage of LPPG by scandium triflate. 2-butanol saturated water (**a**) and phenol-water extracts (**b**) were incubated with increasing concentrations of scandium triflate for 2 hours at 60°C, resulting in increasing degradation of LPPG. Western blot analysis was performed with the anti-LPPG EH5 monoclonal antibody

Further investigation showed that LPPG degradation due to scandium triflate is occurring in a dose-dependent manner. While 5 mM was enough to degrade all LPPG in the previous experiment (Fig. 3a), it was not enough in another 2-butanol saturated water extraction (Fig. 4a). The same was observed with LPPG extracted by phenol-water (Fig. 4b). Figure 4a clearly shows that the dose-dependent degradation of LPPG by scandium triflate ranges from 2 mM (slight degradation) to 10 mM (close to total degradation). The 2-butanol saturated water extract experiences a little downward shift of the 37–55 kDa band to 20–40 kDa in the samples incubated with 4–10 mM scandium triflate. In comparison, the phenol-water extract changes from consisting of two bands to one continuous band (0.5 mM), which even intensifies at 1 mM scandium triflate. At 3 mM and 5 mM scandium triflate, the smear from 40 to 180 kDa diminishes while the lower part (20–40 kDa) remains. At 7 mM, the phenol-water extract has nearly vanished. Thus, LPPG extracted by both methods can be successfully cleaved by approximately 10 mM of scandium triflate.

## Discussion

Extraction of membrane proteins can be achieved by multiple ways depending on their properties. Unfortunately, there is no standard procedure as they constitute a heterologous group of molecules (Smith 2011). LPPG belongs to the lipid-anchored proteins due to the insertion of its GPI-anchor in the cell membrane (Moody-Haupt et al. 2000). The ability to extract and solubilise LPPG is not an easy task, as it is a complex molecule with hydrophilic sugar residues and a hydrophobic, lipid membrane anchor. Classical LPPG extraction methods include the usage of tedious protocols and harmful chemicals. Some methods were adapted from bacterial LPS extraction procedures using hot phenol-water (Westphal and Jann 1965), others from *Leishmania donovani* LPG extraction with solvent E (Turco et al. 1984). Most groups also include a delipidation step based on the lipid extraction method by Bligh and Dyer (Bligh and Dyer 1959). During the preparation of this manuscript, we found that a chemically very different material of similar hydrophobicity as *E. histolytica* LPPG, a lipophosphonoglycan, was extracted from *Acanthamoeba castellanii* using water saturated with 1-butanol (Korn et al. 1974).

In this paper, we investigated the ability of organic solvent mixtures, in particular water mixed with simple alcohols, to extract *E. histolytica* LPPG from the trophozoite membranes in a simple and quick way. We could improve LPPG extraction to a simple and fast protocol (Fig. 1). The western blot analysis with the anti-LPPG antibody EH5 shows that water saturated with 2-methyl-1-propanol, 1-butanol and 2-butanol were the superior solutions for LPPG extraction as most LPPG could be detected in the cell supernatant (Fig. 2a) but not in the pellet (Fig. 2b). Apparently, water saturated with butanol had the ideal hydrophobicity for solubilising LPPG.

Subsequently, we performed further experiments with the 2-butanol saturated water extracted LPPG and, as a comparison, the phenol-water extracted LPPG. Treatment of the LPPG extract with a variety of proteases with different substrate specificities was aimed to generate identifiable protein fragments and to give us insights on the composition of the LPPG protein core. Trypsin, chymotrypsin and proteinase K are all classified as serine proteases but exhibit different cleavage preferences. Trypsin targets amino acid residues with basic side chains, e.g. lysine or arginine, chymotrypsin on the other hand cleaves proteins with aromatic or hydrophobic side chains such as methionine, tryptophan or tyrosine (Rodriguez et al. 2008; Uliana et al. 2021). Proteinase K has broad substrate specificity and is used inter alia for inactivation of nucleases during the isolation of nucleic acids. The results show that incubation of LPPG in 2-butanol saturated water and hot phenol extracts with trypsin and chymotrypsin (Fig. 3a, b, lanes 1–2) led to little to none visible degradation. Proteinase K (Fig. 3a, b, lanes 3) slightly weakened the top bands of LPPG with little changes otherwise. Thermolysin, with a high digestion temperature of 75 °C, theoretically cleaves proteins at the N-terminus of leucine, phenylalanine, valine, isoleucine, alanine and methionine (Feder 1967). In the LPPG in the 2-butanol saturated water extract, only the top bands were slightly reduced by thermolysin (Fig. 3a, lane 4), no digestion was observed in the LPPG extracted by hot phenol (Fig. 3b, lane 4). Incubation of LPPG with the protease from *Aspergillus saitoi* (Fig. 3a, b, lanes 5) resulted in both extracts in partial degradation leading to decreases in molecular mass. On the one hand, this might be attributed to the protease’s β-glucosidase function (Nishinoaki et al. 2008) which is also present in the preparation from Sigma we used. On the other hand, the low pH of 2.8 in the *Aspergillus* protease digestion buffer is not far from the pH 1.7 of the mild acid digestion of LPPG as described before (Bhattacharya et al. 1992b). This could also explain the partial digestion of the LPPG.

Pronase, a non-specific protease mixture from *Streptomyces griseus* (Narahashi et al. 1968) gave the most complex results. In our hands, it was able to degrade a large portion of LPPG (Fig. 3a, 3, lanes 6) as found before (Stanley et al. 1992), but with more complex band patterns. Pronase degradation of the 2-butanol saturated water extract additionally led to the appearance of two bands with a size of approximately 37 kDa (Fig. 3a, lane 6). In comparison, the phenol-water extracted LPPG mainly lost the large bands from 130 to 180 kDa. Although pronase is mainly a protease mixture, it is also known to liquefy mucins (Wesley et al. 1985), which are highly glycosylated glycoproteins like LPPG; however, in this case, pronase probably attacks the protein domains of mucins. In contrast, pronase is even able to digest pure glycans such as chitosan (Kumar et al. 2004). Taken together, important questions remain open about the action of *Aspergillus* protease and pronase on LPPG.

The observation that the classical proteases did not cleave LPPG could, on the one hand, suggest that the sugar side chains of LPPG possibly mask the protein core, thus preventing protease digestion. On the other hand, it could also be conceivable that its protein core consists of amino acid residues other than the recognition sequences of the proteases used. Amino acids not recognised by the proteases are, for example, serine or threonine. The hypothesis of a serine-rich protein core could be supported by LPPG degradation by scandium triflate (Fig. 3a, b, lanes 7). This Lewis acid was shown to cut proteins predominately at serine and threonine residues (Koehler and Thiede 2020). The susceptibility of LPPG to scandium triflate could be explained by multiple cleavages leading to small fragments that are lost during the blotting process resulting in loss of reactivity with antibody EH5.

Based on the hypothesis of a highly serine-rich core protein, the *E. histolytica* HM-1:IMSS database was searched by BlastP (https://blast.ncbi.nlm.nih.gov/Blast.cgi?PAGE=Proteins) for a stretch of 30 serine residues, revealing an unusual gene product (XP_001914036) comprising a predicted amino-terminal signal peptide followed by an uninterrupted stretch of 120 serine residues. This gene product might be hypothesized to be the very unusual LPPG core protein. Although this may be an interesting candidate, so far we cannot provide any evidence supporting this claim.

Mass spectrometric attempts to identify fragments of the core protein via multiple combinations of purification (*e.g.*, filtration, C18*,* non-porous graphitized column, cation exchange columns) and degradation treatments (*e.g.*, scandium triflate, dextranase, phosphoinositide phospholipase C (PI-PLC), hydrofluoric acid (HF), trifluoroacetic acid (TFA)) were unsuccessful. Taken together, so far there is no solid evidence for the existence of a core protein.

Our investigations demonstrate a new method to efficiently extract LPPG from *E. histolytica* by 2-butanol saturated water, which facilitated further research on this important antigen. The additional observations about its resistance to commonly used proteases and in turn, sensitivity to scandium triflate, once more emphasizes the complexity of the LPPG molecule and the challenges to discover its hypothetical Ser- or Thr-rich protein core. Although our work allowed us to identify a candidate, a number of further experiments are needed to support or to abandon this claim.

## Data Availability

The data, mostly western blots, are shown in the manuscript.

## References

[CR1] Ávila EE, Salaiza N, Pulido J, Rodríguez MC, Díaz-Godínez C, Laclette JP, Becker I, Carrero JC (2016). *Entamoeba histolytica* trophozoites and lipopeptidophosphoglycan trigger human neutrophil extracellular traps. PLoS ONE.

[CR2] Barroso L, Abhyankar M, Noor Z, Read K, Pedersen K, White R, Fox C, Petri WA, Lyerly D (2014). Expression, purification, and evaluation of recombinant LecA as a candidate for an amebic colitis vaccine. Vaccine.

[CR3] Bhattacharya A, Ghildyal R, Prasad J, Bhattacharya S, Diamond LS (1992). Modulation of a surface antigen of *Entamoeba histolytica* in response to bacteria. Infect Immun.

[CR4] Bhattacharya A, Prasad R, Sacks DL (1992). Identification and partial characterization of a lipophosphoglycan from a pathogenic strain of *Entamoeba histolytica*. Mol Biochem Parasitol.

[CR5] Bhattacharya A, Arya R, Clark CG, Ackers JP (2000). Absence of lipophosphoglycan-like glycoconjugates in *Entamoeba dispar*. Parasitology.

[CR6] Bligh EG, Dyer WJ (1959). A rapid method of total lipid extraction and purification. Can J Biochem Physiol.

[CR7] Choy SL, Bernin H, Aiba T (2017). Synthetic analogs of an *Entamoeba histolytica* glycolipid designed to combat intracellular *Leishmania* infection. Sci Rep.

[CR8] Diamond LS, Harlow DR, Cunnick CC (1978). A new medium for the axenic cultivation of *Entamoeba histolytica* and other *Entamoeba*. Trans R Soc Trop Med Hyg.

[CR9] Feder J (1967). Studies on the specificity of *Bacillus subtilis* neutral protease with synthetic substrates. Biochemistry.

[CR10] Fehling H, Choy SL, Ting F, Landschulze D, Bernin H, Lender SC, Mühlenpfordt M, Bifeld E, Eick J, Marggraff C, Kottmayr N, Groneberg M, Hoenow S, Sellau J, Clos J, Meier C, Lotter H (2020). Antileishmanial effects of synthetic EhPIb analogs derived from the *Entamoeba histolytica* lipopeptidephosphoglycan. Antimicrob Agents Chemother.

[CR11] Frederick JR, Petri WA (2005). Roles for the galactose-/N-acetylgalactosamine-binding lectin of *Entamoeba* in parasite virulence and differentiation. Glycobiology.

[CR12] Isibasi A, Santa Cruz M, Ramírez A, Kumate J (1982). Immunochemistry of a lipopeptidophosphoglycan extracted from trophozoites of *Entamoeba histolytica* strain HK-9 cultivated in axenic media, using the phenol-water method. Arch Invest Med (mex).

[CR13] Isibasi A, Santa Cruz M, Soto Montano X, Ramírez A, Kumate J (1982). Localization of a lipopeptidophosphoglycan extracted by phenol-water from trophozoites of the HK-9 strain of *Entamoeba histolytica*. Arch Invest Med (mex).

[CR14] Koehler CJ, Thiede B (2020). Predominant cleavage of proteins N-terminal to serines and threonines using scandium(III) triflate. J Biol Inorg Chem.

[CR15] Korn ED, Dearborn DG, Wright PL (1974). Lipophosphonoglycan of the plasma membrance of *Acanthamoeba castellanii*. Isolation from whole amoebae and identification of the water-soluble products of acid hydrolysis. J Biol Chem.

[CR16] Kumar AB, Gowda LR, Tharanathan RN (2004). Non-specific depolymerization of chitosan by pronase and characterization of the resultant products. Eur J Biochem.

[CR17] Leon-Coria A, Kumar M, Chadee K (2020). The delicate balance between *Entamoeba histolytica*, mucus and microbiota. Gut Microbes.

[CR18] Lotter H, González-Roldán N, Lindner B, Winau F, Isibasi A, Moreno-Lafont M, Ulmer AJ, Holst O, Tannich E, Jacobs T (2009). Natural killer T cells activated by a lipopeptidophosphoglycan from *Entamoeba histolytica* are critically important to control amebic liver abscess. PLoS Pathog.

[CR19] Marinets A, Zhang T, Guillén N, Gounon P, Bohle B, Vollmann U, Scheiner O, Wiedermann G, Stanley SL, Duchêne M (1997). Protection against invasive amebiasis by a single monoclonal antibody directed against a lipophosphoglycan antigen localized on the surface of *Entamoeba histolytica*. J Exp Med.

[CR20] Melzer H, Fortugno P, Mansouri E, Felici F, Marinets A, Wiedermann G, Kollaritsch H, von Specht B-U, Duchene M (2002). Antigenicity and immunogenicity of phage library-selected peptide mimics of the major surface proteophosphoglycan antigens of *Entamoeba histolytica*. Parasite Immunol.

[CR21] Moody-Haupt S, Patterson JH, Mirelman D, McConville MJ (2000). The major surface antigens of *Entamoeba histolytica* trophozoites are GPI-anchored proteophosphoglycans. J Mol Biol.

[CR22] Narahashi Y, Shibuya K, Yanagita M (1968). Studies on proteolytic enzymes (pronase) of *Streptomyces griseus* K-1. J Biochem.

[CR23] Nishinoaki M, Asakura T, Watanabe T, Kunizaki E, Matsumoto M, Eto W, Tamura T, Minami M, Obata A, Abe K, Funaki J (2008). Application of an *Aspergillus saitoi* protease preparation to soybean curd to modify its functional and rheological properties. Biosci Biotechnol Biochem.

[CR24] Prasad R, Tola M, Bhattacharya S, Sharma MP, Bhattacharya A (1992). Recognition of *Entamoeba histolytica* lipophosphoglycan by a strain-specific monoclonal antibody and human immune sera. Mol Biochem Parasitol.

[CR25] Rodriguez J, Gupta N, Smith RD, Pevzner PA (2008). Does trypsin cut before proline?. J Proteome Res.

[CR26] Smith SM (2011). Strategies for the purification of membrane proteins. Methods Mol Biol.

[CR27] Srivastava G, Anand MT, Bhattacharya S, Bhattacharya A (1995). Lipophosphoglycan is present in distinctly different form in different *Entamoeba histolytica* strains and absent in *Entamoeba moshkovskii* and *Entamoeba invadens*. J Eukaryot Microbiol.

[CR28] Stanley SL, Huizenga H, Li E (1992). Isolation and partial characterization of a surface glycoconjugate of *Entamoeba histolytica*. Mol Biochem Parasitol.

[CR29] Turco SJ, Wilkerson MA, Clawson DR (1984). Expression of an unusual acidic glycoconjugate in *Leishmania donovani*. J Biol Chem.

[CR30] Uliana F, Vizovišek M, Acquasaliente L, Ciuffa R, Fossati A, Frommelt F, Goetze S, Wollscheid B, Gstaiger M, De Filippis V, auf dem Keller U, Aebersold R,  (2021). Mapping specificity, cleavage entropy, allosteric changes and substrates of blood proteases in a high-throughput screen. Nat Commun.

[CR31] Vines RR, Ramakrishnan G, Rogers JB, Lockhart LA, Mann BJ, Petri WA (1998). Regulation of adherence and virulence by the *Entamoeba histolytica* lectin cytoplasmic domain, which contains a β2 integrin motif. Mol Biol Cell.

[CR32] Wesley A, Mantle M, Man D, Qureshi R, Forstner G, Forstner J (1985). Neutral and acidic species of human intestinal mucin. Evidence for different core peptides. J Biol Chem.

[CR33] Westphal O, Jann K (1965) Bacterial lipopolysaccharides extraction with phenol-water and further applications of the procedure. Methods Carbohydr Chem 5:83–91

[CR34] Zhang Z, Duchêne M, Stanley SL (2002). A monoclonal antibody to the amebic lipophosphoglycan-proteophosphoglycan antigens can prevent disease in human intestinal xenografts infected with *Entamoeba histolytica*. Infect Immun.

